# Etelcalcetide and Paricalcitol in Chronic Kidney Disease: When the Target Is Inflammation

**DOI:** 10.3390/healthcare11010072

**Published:** 2022-12-26

**Authors:** Luis D’Marco, Ana Checa-Ros, Dionilux Gamero, Carlos Soto, Juan Salazar, Manuel Nava, Valmore Bermúdez, Fabiola Dapena

**Affiliations:** 1 Department of Medicine & Surgery, Universidad Cardenal Herrera-CEU, CEU Universities, Carrer Lluis Vives, 1, 46115 Valencia, Spain; 2 Parc Sanitari Sant Joan de Deu, Medicine Department, Carrer Antoni Pujadas, 08830 Barcelona, Spain; 3 Consorci Sanitari del Alt Penedes-Garraf, Nephrology Department, 08720 Barcelona, Spain; 4 Facultad de Ciencias de la Salud, Universidad Simón Bolívar, Barranquilla 080002, Colombia; 5 Endocrine and Metabolic Disease Research Center, School of Medicine, University of Zulia, Maracaibo 4004, Venezuela; 6 Fundación Renal Iñigo Alvarez de Toledo, 28003 Madrid, Spain

**Keywords:** calcimimetics, vitamin D analogs, chronic kidney disease, inflammation

## Abstract

*Introduction:* secondary hyperparathyroidism (SHP) is frequent in patients with chronic kidney disease (CKD), particularly in those in dialysis. To treat this complication, the current options available include phosphorus restriction, phosphate binders, the inhibition of parathyroid hormone (PTH) synthesis and secretion by the supplementation of vitamin D or VDR activators, or the use of calcimimetics. Beyond the control of PTH, the effects of the treatment of SHP on other biomarkers of risk may represent an additional benefit for this population. In this study, we explore the benefits of current SHP treatment options, mainly paricalcitol and/or etelcalcetide in the inflammatory state of hemodialysis (HD) patients. *Results:* the study finally included 142 maintenance HD patients (5 patients were excluded) followed for 6 months (dialysis vintage 26 ± 30 months, mean age 70 years old, 73% women, 81% Spanish white, 47% diabetic). In this case, 52 patients were on regular treatment with paricalcitol for SHP and 25 patients were eligible to initiate etelcalcetide. The baseline serum levels of Ca, P, PTH, Ferritin, albumin, C-reactive protein (CRP), and other variables were measured. We found serum PTH levels showed an improvement after the treatment with etelcalcetide again paricalcitol and no treatment (*p* < 0.04). Of note, serum levels of CRP were significantly lower in a small group of patients (*n* = 11) receiving paricalcitol + etelcalcetide compared to paricalcitol or etelcalcetide alone. The proportion of patients with CRP within target ranges (≤1.0 mg/dL) increased significantly after combined treatment (*p* < 0.001). *Conclusions*: etelcalcetide proved to safely reduce the PTH levels without significant adverse events and the possibility of a synergic anti-inflammatory effect with the simultaneous use of Paricalcitol in HD patients.

## 1. Introduction

Secondary hyperparathyroidism (SHP) is an important complication in patients suffering from chronic kidney disease (CKD), particularly in those receiving renal replacement treatments [1,2]. Elevated serum parathyroid hormone (PTH) contributes to bone and cardiovascular disorders and has been independently associated with all-cause and cardiovascular mortality in CKD patients [3]. The current treatment options for SHP consist of the oral administration of phosphate binders, oral or intravenous (IV) calcitriol, or vitamin D analogs (Paricalcitol), the oral calcimimetic agent Cinacalcet, and the more recently IV Etelcalcetide [4,5]. The treatment with paricalcitol or cinacalcet has been associated with a favorable effect on cardiovascular disease in CKD [6]. On the contrary, although treatment with vitamin D sterols may decrease plasma levels of PTH often raises serum calcium and/or phosphorus concentrations, changes that have been implicated in the development of soft tissue and cardiovascular calcification in patients undergoing dialysis [7]. In contrast, calcimimetic agents such as cinacalcet or etelcalcetide reduce plasma PTH levels while modestly lowering serum calcium and phosphorus concentrations [8].

In patients with CKD, manifestations of cardiovascular disease can be broadly divided into those affecting the myocardium and those affecting the blood vessels. Moreover, these processes are not mutually exclusive. Traditional risk factors for atherosclerotic are insufficient to explain this vastly increased risk. Therefore, the contribution of CKD-specific cardiac risk factors has been postulated including anemia, abnormal bone mineral metabolism, oxidative stress, and chronic inflammation.

Beyond the control of PTH, the effects of the treatment of SHP on other biomarkers of risk may represent an additional benefit in the CKD population. Thus, systemic inflammation plays a major role in the development of atherosclerosis leading to coronary heart disease. C-reactive protein (CRP) is an established marker of systemic inflammation in the general population and patients affected by renal disease. In this study, we explore the benefits of current SHP treatment options, mainly paricalcitol and/or etelcalcetide in the inflammatory state of hemodialysis (HD) patients.

## 2. Materials and Methods

### 2.1. Patients

This was an observational study that evaluated a total population of 147 maintenance HD patients in the dialysis unit of Consorci Sanitari del Alt Penedes Garraf in Barcelona, Spain. The patients that meet the criteria by their nephrologist doctor in charge were eligible to initiate etelcalcetide (HD for ≥3 months with PTH >300 pg/mL, PTH 150–300 pg/mL with calcium-phosphorus product >50 mg^2^/dL^2^ or Cinacalcet intolerance). Additionally, other inclusion criteria were, age ≥18 years at the time of signing informed consent and a life expectancy higher than 1 year. As exclusion criteria, beyond those mentioned in inclusion, patients with an active inflammatory process such as infection, active cancer, or others inflammatory states were excluded. Finally, 142 patients sign their consent to participate (4 patients that reject to sign and 1 patient with active infection were excluded), and the study was approved by the research committee of the hospital.

As a primary outcome, we evaluate the changes in calcium, phosphorus, and PTH serum levels. Additionally, we evaluate the CRP, ferritin, and albumin serum levels as biomarkers of inflammation.

### 2.2. Treatment

The main reason for initiating the treatment with etelcalcetide was inadequate to control of PTH. The starting dose of etelcalcetide was 2.5 mg IV three times weekly (TIW) given at the end of the hemodialysis session. Depending on serum PTH (>300 pg/mL) and calcium (>8.3 mg/dL), the dose was increased by 2.5 or 5 mg at 4-week intervals to a maximum dose of 15 mg TIW. Patients controlled (PTH 150-300 pg/)mL under paricalcitol treatment were kept on the same habitual doses (2 and 5 mg) during the HD sessions. Some patients without PTH control were switched to etelcalcetide or in some cases etelcalcetide was added to the treatment with paricalcitol (those with higher serum PTH levels). Most subjects were receiving stable doses of calcium supplements, phosphate binders, and/or calcitriol or active vitamin D analogs with an albumin-corrected serum calcium of 8.3 mg/dL.

### 2.3. Statistical Analyses

The qualitative variables are shown as absolute and relative frequencies. The chi-square (χ^2^) test was used to determine the association between variables. Quantitative variables are shown as the mean and standard deviation (SD). The Kolmogorov-Smirnov test was applied to check the normality of variable distribution. The Student’s t-test for independent samples or paired samples (as in the case of comparisons of inflammatory markers between baseline and 6 months after treatment) was applied for variables following a normal distribution when there were two groups of comparison. The non-parametrical equivalent (Wilcoxon test) was applied for variables showing a not normal distribution. The SPSS version 19.0 (IBM Corp., Armonk, NY, USA) for Windows was used for all statistical tests. A *p* value < 0.05 was considered significant.

## 3. Results

The patients were followed for 6 months. We finally include 142 maintenance HD patients (dialysis vintage 26 ± 30 months, mean age 70 years old, 73% women, 81% Spanish white, 47% diabetic), 52 patients were on regular treatment with paricalcitol for SHP and 25 patients were eligible to initiate etelcalcetide. The baseline serum levels of Ca, P, PTH, Ferritin, albumin, CRP, and other variables were measured and resumed in Table 1. Most subjects were receiving stable doses of calcium supplements, phosphate binders, and/or calcitriol or active vitamin D analogs.

**Figure 1:**
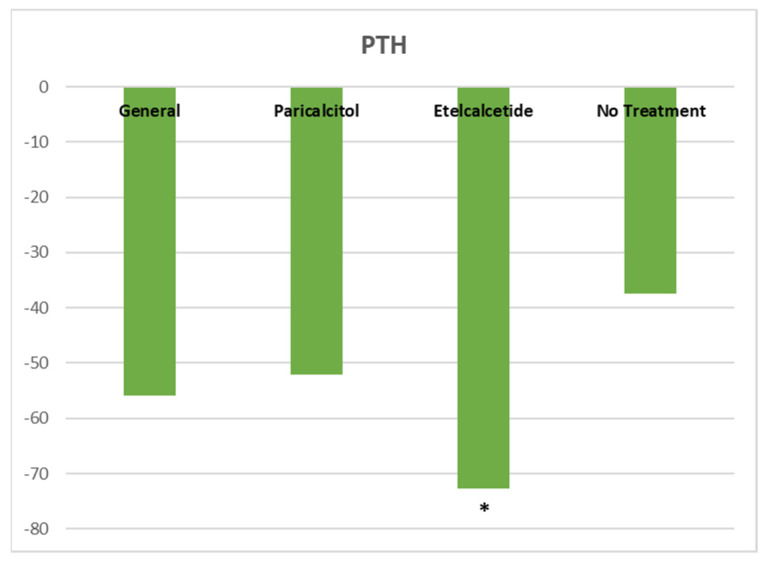
The percentage of decreased PTH values after 6 months of treatment. * PTH % decreased by Etelcalcetide vs. Paricalcitol & no treatment; *p* < 0.04. PTH; Parathyroid hormone.

**Figure 2:**
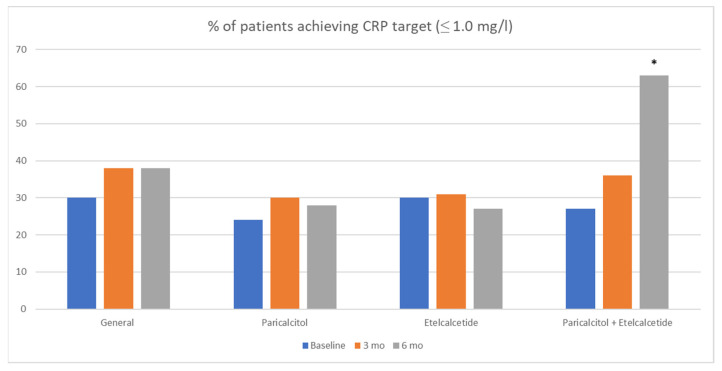
The percentage of patients that reach CRP target values after 6 months of treatment. * CRP at baseline vs. 6 months; *p* < 0.001. CRP; C-Reactive protein. Mo; months.

After the following period, there were no significant changes in Ca, P, and other variables. However, serum PTH levels showed an improvement after the treatment with etelcalcetide. All patients treated with etelcalcetide (*n* = 25) received a starting dose of 2.5 mg TIW after the HD sessions, with a most frequent weekly dose of 10 mg at baseline and at 6-month follow-up, respectively. In the etelcalcetide group, the PTH levels decreased significantly from 743.5 (±502.4) pg/mL at baseline to 371 (±307) pg/mL at month 6 of follow-up (*p* < 0.04), compared to paricalcitol treatment and no treatment **(**Figure 1**)**. In the group receiving paricalcitol, although the PTH levels decreased, do not reach statistical significance compared to those patients without SHP treatment. Calcium and phosphorus levels decreased scarcely in the etelcalcetide group from 9.0 ± 0.9 mg/dL to 8.9 ± 0.4 mg/dL and 5.3 ± 2.2 mg/dL to 4.9 ± 0.3 mg/dL at the end of the study. Of note, serum levels of CRP were significantly lower in a small group of patients (*n* = 11) receiving paricalcitol + etelcalcetide compared to paricalcitol or etelcalcetide alone **(**Table 2). The proportion of patients with CRP within target ranges (≤1.0 mg/dL) increased significantly after combined treatment (*p* < 0.001) (Figure 2). Changes in serum ferritin and albumin levels did not reach statistical significance.

Adverse drug reactions (i.e., events considered related to etelcalcetide) were mainly in those patients with SHP and very high levels of PTH. Asymptomatic hypocalcemia <7.5 mg/dL was observed in 3 cases, which led to a reduced dose of etelcalcetide with subsequent recovery (15 mg to 5 mg/session). One of these patients, treated with both etelcalcetide and paricalcitol, reported gastrointestinal symptoms (abdominal discomfort without nausea).

## 4. Discussion

The key findings of this study were: 1.—Etelcalcetide proved to reduce PTH levels in HD patients with severe SHP; 2.—No significant adverse events were reported; 3.—The possibility of a synergic anti-inflammatory effect with the simultaneous IV uses of paricalcitol and etelcalcetide.

As we comment earlier, for the treatment of SHP the current options include: dietary phosphorus restriction, the use of phosphate binders; the inhibition of PTH synthesis and secretion by the supplementation of calcitriol or other VDR activators (VDRAs), or the use of calcimimetics; and surgical parathyroidectomy, which is a reserved option in refractory cases after pharmacotherapy has failed [9].

The pleiotropic effects of drugs used to control SHP in CKD patients have been reported. Phosphate binders, specifically non-calcium containers as proven to reduce serum uric acid, and inflammation, improve lipids and bone profile, and positive changes in iron-related parameters. In addition, it can attenuate the progression of vascular calcification and epicardial fat deposits in dialysis-dependent patients [10,11,12,13,14].

Investigations have shown that 25-OH-D has pleiotropic effects on the immune system and a possible benefit in patients with chronic inflammatory states [15]. The 25-OH-D deficit has been related to a greater prevalence of cancer and cardiovascular disease [16,17]. Likewise, CKD patients present a truly chronic inflammation state, which plays a major role in the high rate of morbi-mortality events. This uremic-related inflammatory state can be determined by classic biochemical parameters (albumin, ferritin, or CRP). As we comment previously, high CRP levels are a strong predictor of cardiovascular events and mortality. CRP may contribute directly to the pathogenesis of atherosclerosis. It does so by three mechanisms. First, CRP binds to injury cells and activates the complement system. Second, it also displays calcium-dependent in vitro binding and aggregation of low-density lipoprotein and very-low-density lipoprotein. Furthermore, it is a potent stimulator of tissue factor production by monocytes, and the effect is augmented in the presence of other inflammatory mediators [18].

Beyond the control of SHP, a recent study in 45 stable HD patients showed that correcting 25-OH-D deficiency with low doses of oral vitamin D analogs (calcifediol) is associated with an improvement in inflammatory status [19]. Other investigations reported similar results [20,21]. The use of Paricalcitol leads to improve markers that are linked to the progression of CKD. Thus, paricalcitol-induced reduction in albuminuria and inflammation has been reported and, this pleiotropic benefit may be mediated independently of its effects on hemodynamics or PTH suppression [22,23].

Calcimimetics are a novel class of agents for the treatment of SHP in dialysis patients. These agents allosterically bind and activate the calcium-sensing receptor (CaSR), increasing the response of the receptor to serum calcium (orthosteric agonist) [24]. In HD patients with moderate-severe SHP, the ADVANCE study showed that the treatment with cinacalcet in combination with low-dose VDRAs attenuated the progression of vascular and valvular calcification over 52 weeks of following compared to a treatment regimen based on flexible doses of a VDRA alone [25]. Similarly, in the double-blind EVOLVE trial conducted in a large cohort of dialysis patients (*n* = 3.883) with SHP, those treated with cinacalcet on top of standard care showed better control and lower risk of developing severe unremitting SHP compared to the placebo group [26]. Etelcalcetide, a synthetic peptide agonist of the CaSR mainly differs from cinacalcet because it shows mild gastrointestinal symptoms, nausea, and vomiting. In addition to decreasing PTH itself, some studies suggest that etelcalcetide effectively improves bone turnover in patients with CKD undergoing HD with severe SHP [27]. Moreover, lowered fibroblast growth factor 23 (FGF-23), and this reduction may occur via improvements in phosphate and calcium [28]. There is growing evidence showing that etelcalcetide effectively lowered serum PTH, calcium, and phosphate, irrespective of the severity of SHP [27]. Finally, in adenine rat models of kidney disease, etelcalcetide and paricalcitol similarly attenuated the progression of SHP. Nevertheless, etelcalcetide differentially prevented vascular calcification, at least in part, due to reductions in serum FGF-23, calcium, and phosphorus levels [29].

To our knowledge, this is the first report showing that a combination of the etelcalcetide and paricalcitol has a possible synergic benefit effect in chronic inflammation in HD patients. However, our study has several limitations. The most important is the lack of determination of more serum inflammation (IL-6, IL-10, IL-18, TNF-α), bone metabolic (FGF-23 and bone alkaline phosphatase), and uremia markers. Another drawback might be the lack of a more precise measurement of serum CRP using a high-sensitivity assay and the central laboratory measurements of PTH, calcium, phosphate, albumin, and ferritin. Moreover, we did not control dietary phosphate intake or the use of phosphate binders.

## 5. Conclusions

Patients with CKD are at high risk of premature cardiovascular complications. Traditional cardiovascular risk factors do not fully account for the cardiorenal burden in CKD. Thus, nontraditional risk factors such as uremia and chronic inflammation impact the unique cardiovascular risk burden of patients with CKD. In conclusion, as the first intravenous calcimimetic, etelcalcetide has proved its safety and efficacy in randomized clinical trials, although the real-world experience reminds limited. Even though a direct association or synergic effects of the SHP treatment beyond PTH control has not been reported with the combination of etelcalcetide and paricalcitol, the significant decrease of CRP appears to support the notion that the chronic inflammation state in HD patients could be improved. Finally, long-term randomized and controlled trials are required to confirm these benefits for renal-affected patients.

## Figures and Tables

**Table 1:** The baseline of the general and other groups with analytical characteristics of the patients.

Variable	Total (*n* = 142)		Paricalcitol (*n* = 52)		Etelcalcetide (*n* = 25)		Paricalcitol + Etelcalcetide (*n* = 11)	
	Mean/%	SD	Mean/%	SD	Mean/%	SD	Mean/%	SD
Age (year)	70	7	72	9	67	7	64	6
Women	73%	-	52%	-	67%	-	76%	-
Race: Spanish White	81%	-	70%	-	80%	-	90%	-
Diabetes	47%	-	52%	-	61%	-	88%	-
Hypertension	42%	-	70%	-	84%	-	82%	-
Dialysis vintage (months)	26	30	30	18	38	15	36	12
Serum Hb (gr/dL)	11.3	12.9	11.1	4.7	11.9	2.1	11.5	1.1
Serum PTH (pg/mL)	362.1	364.0	356.7	440	743.5	502.4	862.9	402.4
Serum Calcium (alb-corrected) (mg/dL)	8.4	0.6	8.7	2.3	9.0	0.9	8.9	0.6
Serum Phosphate (mg/dL)	4.5	1.1	4.3	3.1	5.3	2.2	5.5	2.1
Serum Albumin (gr/L)	3.3	0.3	3.4	0.4	3.4	0.3	3.3	0.2
Serum Magnesium (mg/dL)	2.2	0.3	2.1	0.2	2.4	0.4	2.4	0.3
Serum Phosphatase alkaline (IU/L)	106.2	58.6	110.3	70.1	114.1	28.3	102.1	41.3
Serum CRP (mg/dL)	1.1	1.4	1.3	1.8	1.1	1.9	1.7	2.1
Serum Ferritin (mg/dL)	767.9	447.0	767.2	411.1	899.7	79.3	803.5	98.1
Vitamin D (ng/mL)	12.6	5.3	12.1	7.3	13.3	4.8	8.2	7.1
Use of vitamin D sterols	57%	-	64%	-	69%	-	83%	-
Use of phosphate binders	79%	-	85%	-	92%	-	94%	-

SD, standard deviation; HB; hemoglobin; PTH; parathyroid hormone; Alb; albumin; CRP; c-reactive protein.

**Table 2:** The changes of inflammatory serum biomarkers at baseline and after 6 months of treatment.

Treatment	Ferritin (mg/dL)				Albumin (gr/L)				CRP (mg/dL)			
	Baseline (m)	SD	6 mo (m)	SD	Baseline (m)	SD	6 mo (m)	SD	Baseline (m)	SD	6 mo (m)	SD
**General population**	767.9	447.0	763.8	424.3	3.3	0.3	3.4	0.3	1.1	1.4	1.4	1.4
**Paricalcitol**	767.2	411.1	824.3	398.5	3.4	0.4	3.3	0.3	1.2	1.8	1.5	1.4
**Etelcalcetide**	899.7	79.3	802.2	83.3	3.4	0.3	3.4	0.4	1.0	1.9	1.2	1.5
**Etelcalcetide + Paricalcitol**	803.5	98.1	775.4	88.7	3.3	0.2	3.3	0.4	1.7	2.1	1.1 *	1.3

The values are represented by mean and SD. * (CRP changes at Baseline vs. 6 months; *p* < 0.04). CRP; C-Reactive protein. Mo; months.

## Data Availability

Data are available upon request.
